# Death of female flower microsporocytes progresses independently of meiosis-like process and can be accelerated by specific transcripts in *Asparagus officinalis*

**DOI:** 10.1038/s41598-019-39125-1

**Published:** 2019-02-25

**Authors:** Mayui Ide, Kiyoshi Masuda, Daisuke Tsugama, Kaien Fujino

**Affiliations:** Laboratory of Crop Physiology, Research Faculty of Agriculture, Hokkaido University Kita 9 Nishi 9 Kita-ku, Sapporo-shi, Hokkaido, 060-8589 Japan

## Abstract

*Asparagus officinalis* (garden asparagus) is a dioecious perennial crop, and the dioecy (i.e., sex) of *A. officinalis* can affect its productivity. In *A. officinalis*, flower anthers in female plants fail to accumulate callose around microsporocytes, fail to complete meiosis, and degenerate due to cell death. Although 13 genes have been implicated in the anther development of male and female flowers, it is unclear how these genes regulate the cell death in female flower anthers. The aim of this study was to narrow down factors involved in this process. TUNEL staining and Feulgen staining of female flower microsporocytes suggest that female microsporocytes enter a previously undetected meiosis-like process, and that the cell death occurs independently of this meiosis-like process, excluding the possibility that the cell death is caused by the cessation of meiosis. RNA sequencing with individual floral organs (tepals, pistils and stamens) revealed that several genes possibly regulating the cell death, such as metacaspase genes and a Bax inhibitor-1 gene, are differentially regulated between female and male flower anthers, and that genes involved in callose accumulation are up-regulated only in male flower anthers. These genes are likely involved in regulating the cell death in female flower anthers in *A. officinalis*.

## Introduction

**Figure 1:**
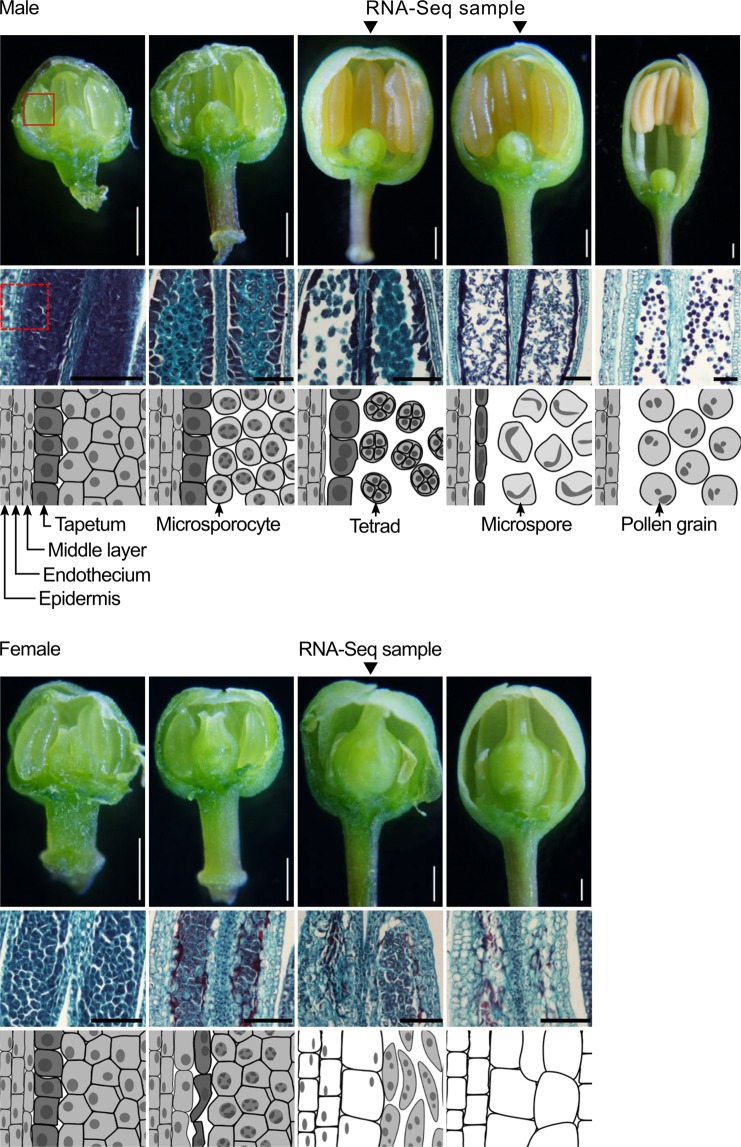
Development of *A. officinalis* flowers. Male and female flowers were collected at the indicated postmeiotic stages. Individual floral organs of these flowers were subjected to RNA-Seq. In both the top panel (male flowers) and bottom panel (female flowers), the middle images show the patterns of safranin-fast green staining in longitudinal sections of anthers. For example, the rectangle with a solid line in the top left image in the top panel corresponds to the field shown in the middle image. The bottom images show the associated states of cells in anthers. For example, the rectangle with a broken line in the middle left image in the top panel corresponds to the field shown in the bottom image. White scale bars for the top images = 0.5 mm; black scale bars for the middle images = 0.1 mm.

Nearly 90% of angiosperms develop hermaphroditic flowers, which have both pistils and stamens^1^. In many hermaphroditic species, pistils and anthers are in close proximity to allow self-pollination, but in others, pistils and anthers separated to promote outcrossing^2^. The rest (~10%) of the angiosperms develop unisexual (i.e., female or male) flowers, where either stamens or pistils are sterile, respectively. Individuals of monoecious plant species develop both female and male flowers, whereas individuals of dioecious species develop only female flowers or male flowers. *Asparagus officinalis* (garden asparagus) is a dioecious crop, and the *A. officinalis* dioecy is important because male plants are preferred to female plants in open-field cultivation of *A. officinalis*. (This is because female plants develop and drop seeds, the offspring of which compete for nutrients with older plants and cause trouble in field management.) In *A. officinalis* female flowers, anthers degenerate due to cell death. The cell death in female flowers occurs first in tapetal cells, which surround microsporocytes to help their development, then in microsporocytes at a stage when male flower microsporocytes would accumulate callose and complete meiosis, and finally in other cells^3^ (see also Fig. 1). It is unclear whether or not the death of these cells is promoted only by defects in tapetal cells, callose accumulation and/or meiosis.

A pair of chromosomes that determine sex is called sex chromosomes. Sex chromosomes of some dioecious species such as *Silene latifolia* are different in size and structure^4^, but the *A. officinalis* sex (*X* and *Y*) chromosomes are morphologically indistinguishable from each other^5^. In *A. officinalis*, plants with the *XX* genotype are female, and either *XY* or *YY* plants are male. The *YY* males, which are called supermales, can be obtained by either culturing male flower anthers^6^ or selfing a hermaphroditic individual if one can be found^7^, and are used to generate “all-male” seeds (i.e., *XY* (male) seeds derived from crosses between *XX* and *YY*). The *A. officinalis Y* chromosome has the *M* locus, which contains a small number of genes that promote masculinization (i.e., that determine the *A. officinalis* sex). Genome sequencing and RNA sequencing (RNA-Seq) have identified 13 *M* locus gene candidates, and two of them, *SOFF* (*SUPPRESSOR OF FEMALE FUNCTION*) and *AoMYB35* (*A. officinalis MYB35*, also known as *MSE1* (*MALE-SPECIFIC EXPRESSION 1*) and *AspTDF1* (*Asparagus DEFECTIVE IN TAPETUM DEVELOPMENT AND FUNCTION 1*)) are thought to be especially important^8–12^. *SOFF* is present only in the male genome in *A. officinalis*, encodes a protein with a domain of unknown function (DUF247), and suppresses pistil development in male flowers with unknown mechanisms^12^. *AoMYB35* is the putative orthologue of *Arabidopsis thaliana MYB35* (*AtMYB35*), is present only in the male genome, and is expressed in male flower stamens at a premeiotic stage^9,10^. *AtMYB35*-knockout (*atmyb35*) plants have tapetal cell dysfunctions and develop no microspores^13^. Thus, the defects in the anther development in *A. officinalis* female flowers are likely, at least partially, due to the lack of *AoMYB35*. However, unlike anthers of *A. officinalis* female flowers, *atmyb35* anthers stay undegenerated and accumulate callose around microsporocytes^13^. In addition, three of seven dioecious *Asparagus* species other than *A. officinalis* (*A. acutifolius*, *A. stipularis* and *A. cochinchinensis*) were reported to have an *MYB35*-like gene in both males and females, raising the possibility that the *MYB35*-like genes are not direct regulators of the dioecy in these species^9,12^. Precise functions of *AoMYB35* are therefore unknown. Further characterization of the cell death in female flower anthers can help to identify factors that act with or downstream of the 13 *M* locus gene candidates.

Here, to narrow down the factors that regulate the *A. officinalis* anther development, female and male flowers are further characterized. Histological analyses exclude the possibility that the cell death in female flower microsporocytes is caused by the cessation of meiosis, and support the idea that female flower microsporocytes have defects in the cell wall, possibly due to callose deficiency. Transcriptomes analyses suggest that genes involved in the cell death and the callose accumulation in anthers are differentially regulated between female and male flower anthers.

## Results and Discussion

### Histological characterization of cell death in anthers in female flowers

**Figure 2:**
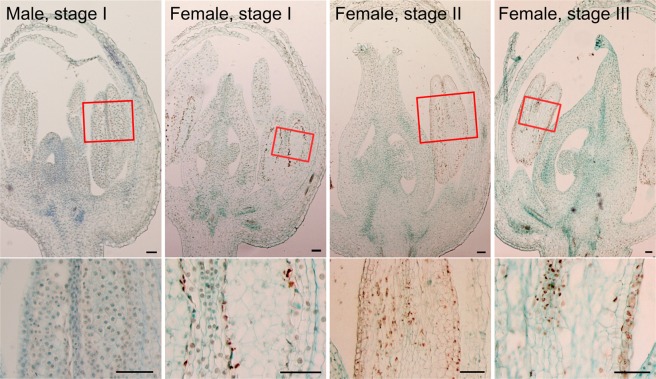
TUNEL staining of female and male *A. officinalis* flowers. Brown signals correspond to the TUNEL signals, whereas blue signals correspond to the Fast Green FCF counterstaining signals. Relative developmental stages (I-III) are shown in each panel. Male flowers sampled at stages II and III became fragile after TUNEL staining, and did not show reproducible patterns of staining. The bottom images are magnified images for the regions in rectangles in the top images. Scale bars = 50 μm.

Maize (*Zea mays*) and cucumber (*Cucumis sativus*) are monoecious species that use cell death to develop unisexual flowers, and DNA is degraded in the process of cell death in these species^14,15^. However, it is unclear whether such DNA fragmentation occurs in death of cells in *A. officinalis* female flower anthers. To further characterize the death of these cells, DNA fragmentation was analyzed by TUNEL (terminal deoxynucleotidyl transferase-mediated dUTP nick end labeling) staining. In *A. officinalis* female flowers in an early premeiotic stage, no TUNEL staining was detected (Supplementary Fig. S1). At a stage corresponding to the meiosis-initiation stage, while hardly any TUNEL staining was detected in male flowers (Fig. 2, left panel), strong signals were detected in tapetal cells in female flowers (Fig. 2, second from the left). (At later stages, TUNEL staining made male flowers fragile, resulting in irreproducible staining patterns.) The patterns of TUNEL staining were more stable in female flowers than in male flowers, and at a putative meiotic stage, strong signals were detected in microsporocytes and cells in the endothecium and the middle layer in female flowers (Fig. 2, third from the left). At a later stage when the death of tapetal cells and microsporocytes seemed completed, strong signals were detected in epidermal cells of the anthers (Fig. 2, right). These results support the idea that DNA fragmentation is a part of the process of cell death in female flower anthers of *A. officinalis*.

**Figure 3:**
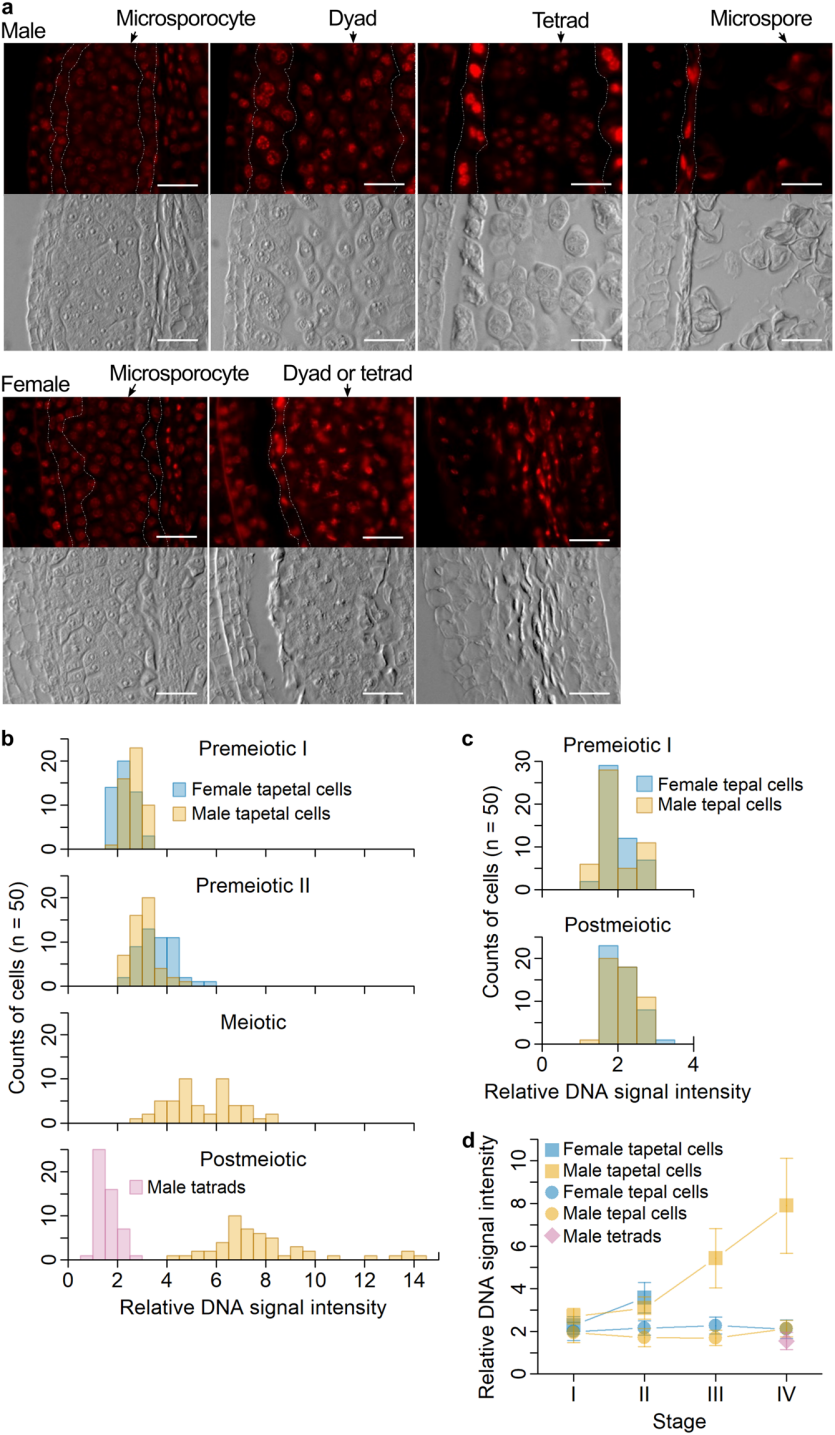
Characterization of DNA states in tapetal cells by Feulgen staining. (**a**) Signals of Feulgen staining (top images for both the top panel for male flowers and the bottom panel for female flowers) and differential interference contrast images of the same cells (bottom images). Boundaries of the tapetum are indicated by white, broken lines. Scale bars = 25 μm. (**b**) Histograms of Feulgen staining intensity in tapetal cells. For the postmeiotic stage, the histogram for male tetrads, which are haploids, is shown as a control. (**c**) Histograms of Feulgen staining intensity in tepal cells. (**d**) Line plots of Feulgen staining intensity. The stages I-IV correspond to the stages “Premeiotic I”, “Premeiotic II”, “Meiotic”, and “Postmeiotic” in the panel b, respectively. Data are means ± uncorrected sample standard deviations for 50 cells.

Previous electron microscopy showed that the tapetal cells in *A. officinalis* male flowers become binucleate at a premeiotic stage but those in female flowers do not^3^. Feulgen staining allows semi-quantification of DNA, and was used to further examine DNA states before and after meiosis in *A. officinalis* anther cells. In agreement with the previous finding^3^, in tapetal cells in male flowers, only one strong, circular signal of Feulgen staining was observed at a premeiotic stage and two were observed at the meiotic stages (Fig. 3a, upper images). Also in agreement with the previous finding^3^, only one circular signal was observed in a tapetal cell in female flowers even at a later developmental stage when such a cell was deformed (Fig. 3a, lower images). These results confirm that tapetal cells in male flowers become binucleate at the meiosis-initiation stage and that tapetal cells in female flowers stay uninucleate until the cell death process is completed. The intensity of Feulgen staining gradually increased in tapetal cells in male flowers, and was more than two times stronger at the postmeiotic stage than at the premeiotic stage. The signals also slightly increased in tapetal cells in female flowers until they degenerated. Thus, DNA synthesis in these cells might be partially activated, although they do not become binucleate. No such increases in signals of Feulgen staining were observed in tepal cells in either male or female flowers (Fig. 3b-d).

**Figure 4:**
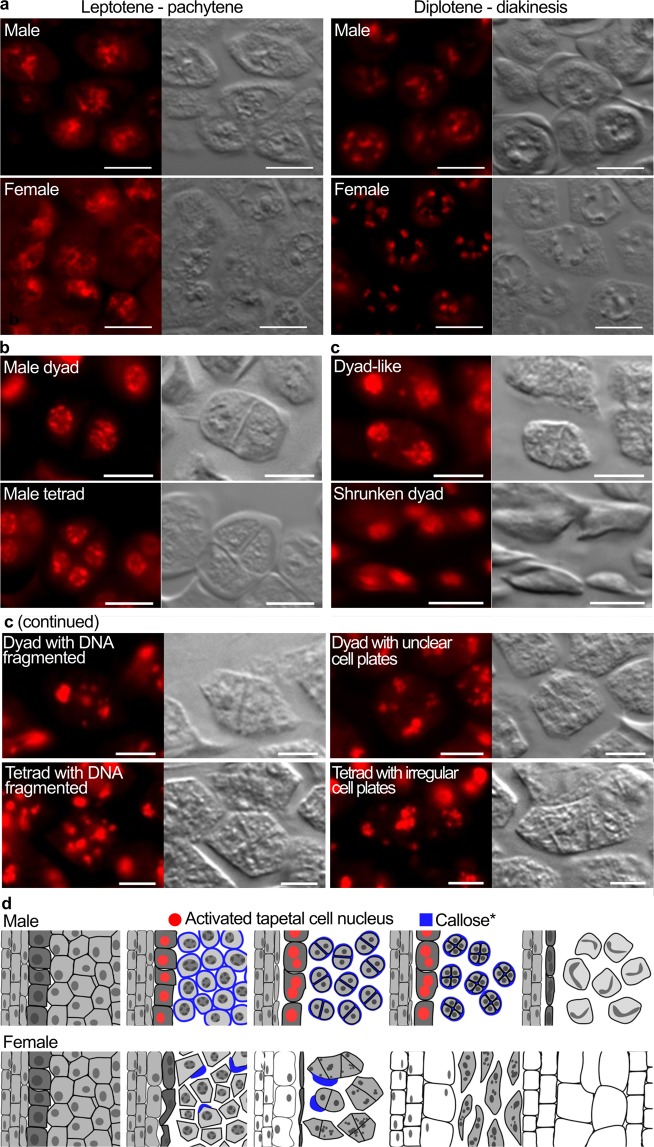
Characterization of cell and DNA states in microsporocytes by Feulgen staining and differential interference contrast (DIC) microscopy. For panels a-c, Feulgen staining is shown in the left, and DIC images of the same cells are shown in the right, and all the scale bars = 10 μm. (**a**) DNA in fibrous (left panels) and condensed forms (right). The putative phases of meiosis are indicated. (**b**) Typical appearances of a dyad (top) and a tetrad (bottom) in male flowers. (**c**) Female flower microsporocytes with different extents of cell deformation and DNA fragmentation. (**d**) Schematic representation of pollen maturation in male flowers (top) and defects in pollen maturation in female flowers (bottom). *: callose accumulation patterns are based on previous studies^5,12^.

Female flower microsporocytes hardly accumulate callose around them^3,10^ and do not complete meiosis^3^, but their DNA is condensed at a stage corresponding to the meiosis-initiation stage^10^. Feulgen-stained DNA in female flower microsporocytes became fibrous and then condensed in a synchronized manner within a locule, just as DNA in male flower microsporocytes did at premeiotic stages (Fig. 4a). After these stages, the number of strong, circular signals of Feulgen staining in a male flower microsporocyte became two, then four, and putative cell plates were visible between these signals (Fig. 4b), suggesting that dyads and tetrads were formed. In contrast, although some female flower microsporocytes also showed two strong signals of Feulgen staining separated with a cell plate (Fig. 4c, top left), such signals were often deformed. Some shrunken microsporocytes also showed two strong, circular signals of Feulgen staining (Fig. 4c, bottom left). In many other microsporocytes in female flowers, many smaller, yet strong, signals of Feulgen staining with irregularly oriented cell plates were observed (Fig. 4c, the rest). These results suggest that female flower microsporocytes partially undergo a process similar to meiosis, and that during this process, they become deformed and their DNA is fragmented. Tetranucleate microsporocytes were rarely observed in female flowers (Fig. 4c), raising the possibility that the process of cell death rapidly progresses immediately after the first division in the meiosis-like process in female flowers. However, because the meiosis-like process in female flower anthers should progress in a synchronized manner within a locule, as the meiosis in male flower anthers does, and because the extents of cell deformation and DNA fragmentation are not uniform between microsporocytes within a locule in a female flower (Fig. 4c), the progress of cell death should be independent of the progress of the meiosis-like process. Cell shape can be determined by cell wall structure and callose is a component of the cell wall. The callose deficiency in female flower microsporocytes^5,12^ may therefore be partly responsible for the deformation and/or the irregular formation of cell plates in these cells. These previous findings about callose and new findings about DNA states during cell death are summarized in Fig. 4d.

### Validation of RNA-Seq with individual floral organs of *A. officinalis*

**Figure 5:**
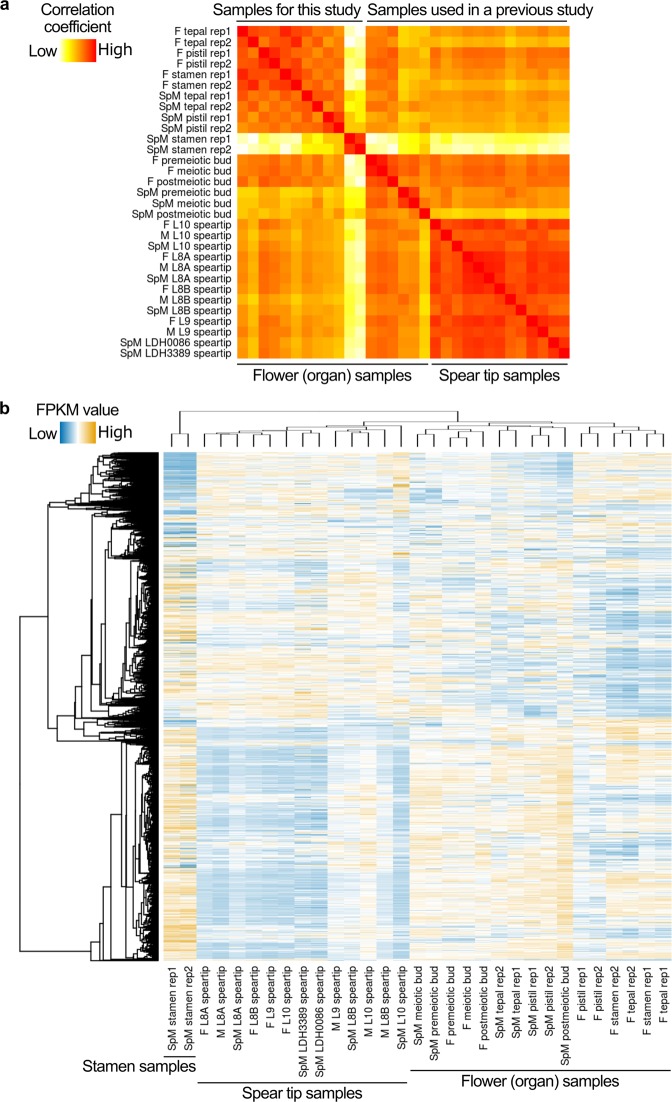
Characterization of samples used for RNA-Seq. (**a**) Heat map based on Pearson’s correlation coefficients. Raw values of the correlation coefficients were used for coloration (see Supplementary Table S2 for exact values). The orders of samples are the same between rows and columns, and sample names for columns are omitted. F: female; M: male; SpM: supermale; L: line; rep1: replicate 1; rep2: replicate 2 (these also apply to the panel b). (**b**) Heat map and hierarchical clustering for genes with FPKM values higher than 100. Manhattan distances of log_10_-transformed FPKM values were used to cluster genes with the group average method^42^ and to cluster samples with Ward’s method^43^. Dendrograms for gene clustering and sample clustering are presented in the left (i.e., horizontally) and the top (vertically), respectively. Gene names for rows are omitted. Normalized log_10_-transformed values were used for coloration.

Tepals, pistils and stamens of female and supermale *A. officinalis* plants were collected at postmeiotic stages, when the degeneration of female flower anthers and the cessation of male flower pistils were almost complete (see Fig. 1), and subjected to RNA-Seq. Two biological replicates were made for each sample. For each replicate for each sample, 3.3–5.2 Gb data were obtained (Supplementary Table S1). The obtained reads were mapped to the RNA-Seq-derived contigs^8^ rather than the recently published *A. officinalis* genome sequence^12^. This was because this genome sequence still has many regions with undetermined (“N”) bases and because in our MegaBLAST analysis^16^, approximately 2% (5309 out of 276556) of the RNA-Seq-derived contigs (hereafter referred to as “genes”) were not mapped to the genome sequence, raising the possibility that the genome sequence did not cover all the genes present. From the mapping results, FPKM (fragments per kilobase of exon per million mapped fragments) values, which could be regarded as normalized expression levels, were obtained for each gene. In a previous study, FPKM values were obtained in a similar way for floral buds in premeiotic, meiotic and postmeiotic stages, and for speartips^10^, and they were also used for this study. The Pearson’s correlation coefficients for these FPKM values were low for any combinations including the male flower stamen sample, while they were rather high for combinations between the other floral organ samples and between spear tip samples. (Fig. 5a for a heat map; Supplementary Table S2 for exact correlation coefficients). Similar results were obtained in hierarchical clustering (Fig. 5b). These results are consistent with previous findings that transcriptomes in pollen grains are largely different from transcriptomes in vegetative tissues in Arabidopsis^17,18^. Previously, some of the genes that possibly regulate *A. officinalis* anther development were expressed in male floral buds but not in female floral buds^8^. The transcriptome in female flower stamens was more similar to transcriptomes in tepals and pistils than to the transcriptome in male flower stamens (Fig. 5). These results suggest that not only histological changes but also transcriptional changes essentially do not occur in *A. officinalis* female flower microsporocytes.

**Figure 6:**
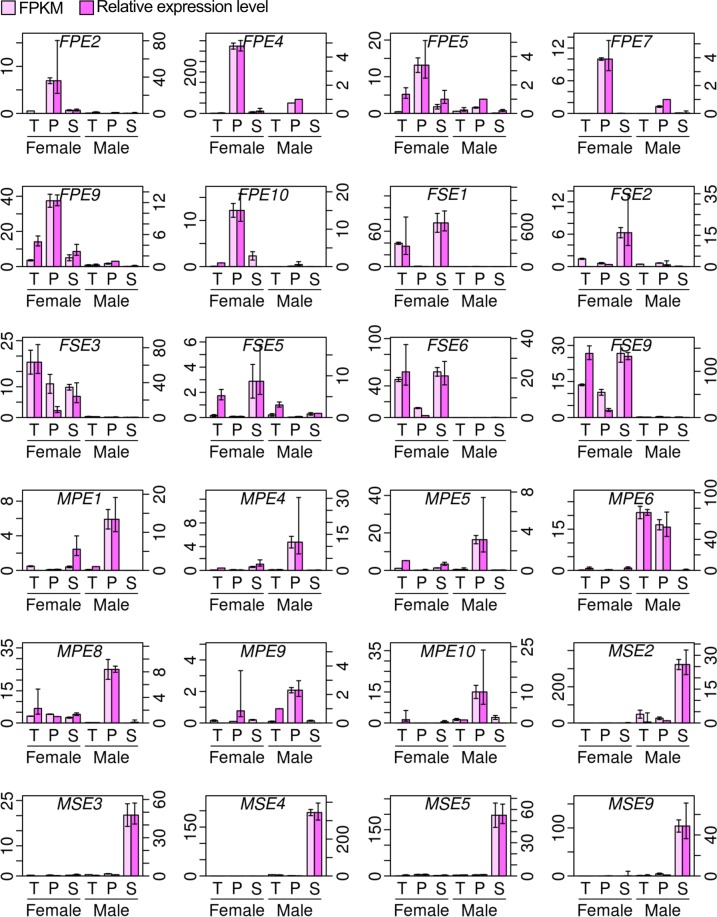
RT-PCR analyses of expression levels of genes with FPKM values higher in pistils and stamens. For each panel, y axes in the left and right indicate the FPKM value and the relative expression level, respectively. FPKM values are presented as means ± uncorrected sample standard deviations of two replicates. Relative expression levels were calculated with the comparative cycle threshold method using a gene encoding ubiquitin-ribosomal peptides as the internal control, and are presented as means ± uncorrected sample standard deviations of three biological replicates. T: tepal; P: pistil; S: stamen. Samples from supermale flowers are referred to as “Male” in this figure. Exact names and possible functions of these genes are available in Supplementary Table S3.

Using the floral organs sampled in the same way as those for the RNA-Seq, reverse transcription-PCR (RT-PCR) was performed to confirm the expression levels of 24 genes that exhibited either female-biased or male-biased expression in pistils and/or stamens in RNA-Seq (see Supplementary Table S3 for their possible functions). The RT-PCR-based expression levels of these genes were consistent with the FPKM values (Fig. 6).

### Transcriptomes of individual floral organs of *A. officinalis*

On the basis of FPKM values, the other sex-dependently expressed genes (SDEGs) were extracted (see the “Analysis of RNA-Seq data” subsection for the “Methods” section for the criterion; Supplementary Table S4 for the extracted genes). Jaccard indices obtained from these sets of SDEGs were somewhat consistent with the Pearson’s correlation coefficients described above, but not higher than 0.2 in most combinations of samples (Supplementary Fig. S2 for heat maps; Supplementary Table S5 for exact Jaccard indices), suggesting that those samples do not share many SDEGs. *AoMYB35* (gene ID: Aspof_comp61397_c0_seq. 6) was included in the SDEGs for premeiotic supermale floral buds but not in those for supermale flower stamens (Supplementary Table S4). This is probably because the supermale flower stamens were sampled at a meiotic and/or postmeiotic stage. *SOFF* consists of three fragmentary contigs: Aspof_comp63234_c1_seq. 5, Aspof_comp63232_c0_seq. 5 and Aspof_comp64490_c0_seq. 2. None of these contigs was included in SDEGs for any sample (Supplementary Table S4; see Supplementary Table S6 for FPKM values for these contigs). This might be because *SOFF* is expressed only at specific stages or in specific cell types.

Arabidopsis proteins have been well-annotated, and associated with many gene ontology (GO) terms^19^ and metabolic pathways. On the basis of similarities between Arabidopsis proteins and the proteins potentially encoded by *A. officinalis* genes, GO terms and metabolic pathways in the AraCyc database^20^ were associated with *A. officinalis* genes. Pistils in *A. officinalis* male flowers substantially differ in shape from pistils in hermaphroditic plants, and stamens in *A. officinalis* female flowers differ from stamens in hermaphroditic plants. GO terms associated to these organs included the terms regarding cell death and phytohormone responses, which should play versatile roles in plants (Supplementary Tables S7 and S8; Supplementary Fig. S3). For the SDEGs for stamens in female flowers, it was notable that the terms related to the photosystems I and II in the thylakoid membrane were overrepresented (Supplementary Fig. S3). These photosystems can produce reactive oxygen species (ROS) as byproducts of photosynthetic electron transfer^21,22^, and such ROS can trigger cell death^23^. Although the level of chlorophyll, which is a major photoreceptor and initiates photosynthetic electron transfer, should be low in stamens in female flowers, the presence of components of the photosystems I and II might promote ROS production and subsequent cell death. It is also possible that phytohormones contribute to such processes.

### Genes possibly regulating cell death are sex-dependently expressed

**Figure 7:**
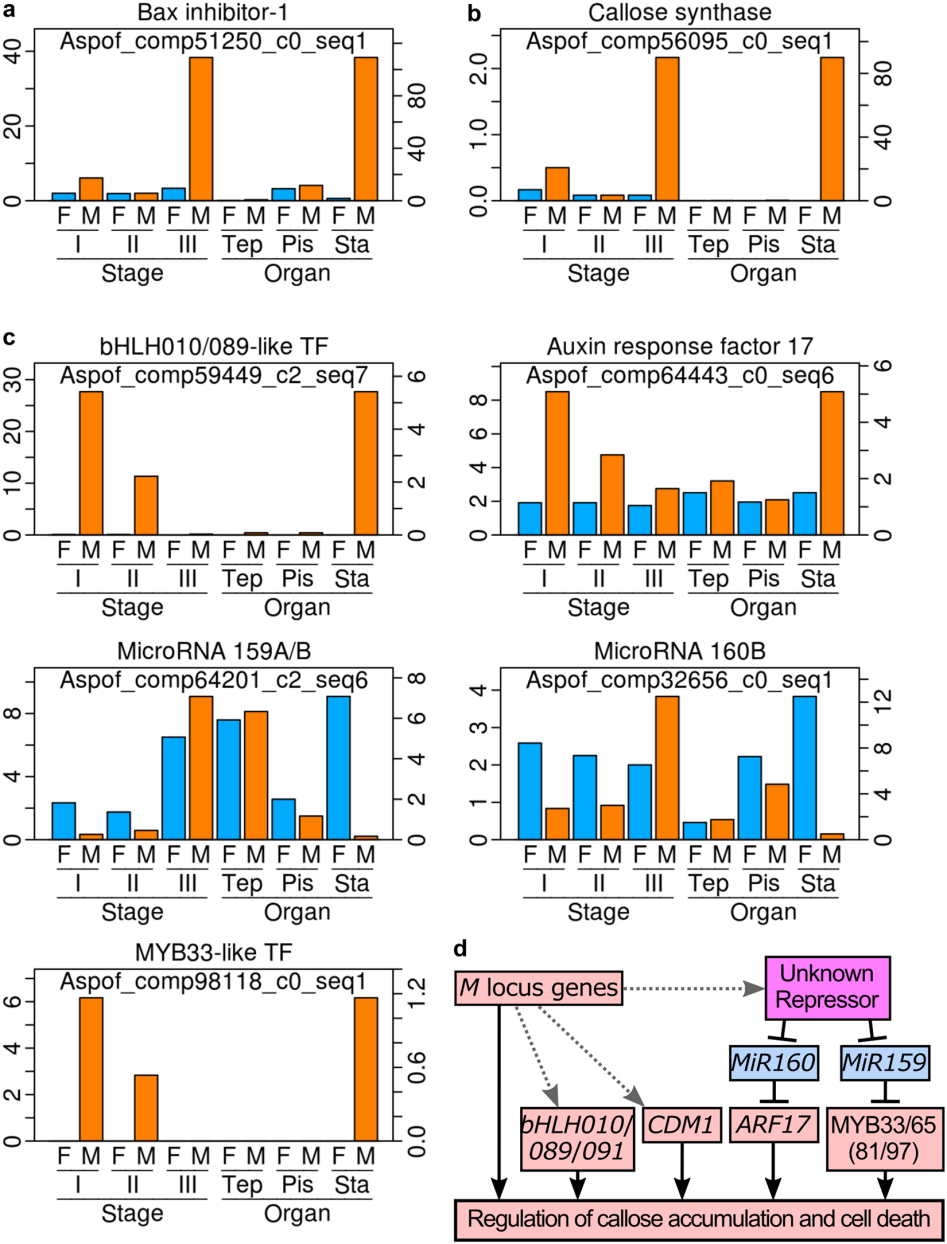
Genes possibly regulating *A. officinalis* anther development. For the panels a-c, the FPKM values for the female (F) and male (M) flower buds at premeiotic (I), meiotic (II) and postmeiotic (III) stages were obtained using previously published RNA-Seq data^8^, and are shown with the scale in the left side in each panel. The FPKM values for tepals (Tep), pistils (Pis) and stamens (Sta) in female (F) and male (M) flowers were obtained in this study, and are shown with the scale in the right side in each panel. Functional annotations indicated on the top of the panels a-c are based on the Arabidopsis homologue most similar to the corresponding *A. officinalis* gene (see Supplementary Table S9 for accession numbers of Arabidopsis genes). TF: transcription factor. (**a**) FPKM values of a Bax inhibitor-1 gene. (**b**) FPKM values of a callose synthase gene. (**c**) FPKM values of transcription factor genes and microRNAs. (**d**) Relationship between *M* locus genes and other transcription factor genes that are likely relevant to *A. officinalis* anther development.

Caspases and Bax inhibitor-1 (BI-1) proteins are inducers and inhibitors, respectively, of programmed cell death in animals, and their homologues are thought to regulate plant cell death as well. For example, caspase homologues (metacaspases) are essential for embryogenesis of Norway spruce^24^. Microspore development is aborted when death of tapetal cells is inhibited at the tetrad stage by artificial ectopic expression of a BI-1 homologue in Arabidopsis^25^. In RNA-Seq, a metacaspase gene was found to be strongly expressed in male flower stamens at any stage, whereas another metacaspase gene was strongly expressed in female flower stamens at postmeiotic stages (Supplementary Fig. S4a, top panels). Some DNA/RNA endonuclease genes were also strongly expressed in sex-, stage- and floral organ-specific manners (Supplementary Fig. S4a, bottom). A BI-1 gene was strongly expressed in male flower stamens, but not in female flower stamens at the postmeiotic stage (Fig. 7a). The differential expression of these cell death-related genes may accelerate the cell death and/or the DNA degradation in female flower microsporocytes. Callose synthase and UDP-glucose pyrophosphorylase are involved in callose synthesis^26,27^, and two *A. officinalis* genes possibly encoding these proteins were strongly expressed in male flower stamens but not in female flower stamens (Fig. 7b for the callose synthase gene; Supplementary Fig. S4b, top panel, for the UDP-glucose pyrophosphorylase gene). Some glycosyl hydrolases are thought to degrade callose^28^, and two *A. officinalis* genes possibly encoding these proteins were also strongly expressed only in male flower stamens (Supplementary Fig. S4b, bottom panels). These results suggest that the callose deficiency in female flower anthers is caused by the decreased expression of the callose biosynthesis genes.

DYSFUNCTIONAL TAPETUM1 (DYT1) is a basic helix-loop-helix (bHLH) transcription factor that can induce three genes encoding closely related bHLH transcription factors, bHLH010, bHLH089 and bHLH091, which are required for callose accumulation around microsporocytes, in Arabidopsis^29,30^. Although the expression of the *DYT1* homologue in *A. officinalis* was weak in all the samples in RNA-Seq, expression of homologues of *bHLH010*, *bHLH089* and *bHLH091* was all strong in male floral buds at the premeiotic stage (Supplementary Fig. S4c for two of them; Fig. 7c for the other). AUXIN RESPONSE FACTOR17 (ARF17) and CALLOSE DEFECTIVE MICROSPORE1 (CDM1) are two other transcription factors required for callose accumulation around microsporocytes in Arabidopsis^28,31^. Expression of homologues of *ARF17* and *CDM1* in *A. officinalis* was also strong in male floral buds at the premeiotic stage, and male flower stamens at a later stage (Supplementary Fig. S4c for the *CDM1*-like gene; Fig. 7c for the *ARF17*-like gene). *ARF17* can be silenced by *microRNA160* (*miR160*) in Arabidopsis^32^, and the *miR160* homologue was strongly expressed in female flower stamens in *A. officinalis* (Fig. 7c). *MYB33* and *MYB65* are MYB transcription factor genes that also regulate tapetum development and are silenced by *microRNA159* (*miR159*) in Arabidopsis^33^. Homologues of *MYB33* and *MYB65*, as well as their two close homologues, *MYB81* and *MYB97*, were all strongly expressed in male flower stamens in *A. officinalis*, and consistent with this, the expression of the *mir159* homologue was strong only in female-derived samples (Fig. 7c for the *MYB33*-like gene and *mir159*; Supplementary Fig. S4c for *MYB65*-like, *MYB81*-like and *MYB97*-like genes). The expression levels of these transcription factor genes and microRNAs were not completely consistent between the stage III (postmeiotic) samples and the isolated stamen samples (Fig. 7c and Supplementary Fig. S4c), possibly because the isolated stamen samples had been contaminated with stamens at the meiotic stage (or stage II). Nevertheless, together, these results support the idea that the sex-dependent expression of the cell death-related genes and the callose-related genes is caused by the male-biased expression of these transcription factor genes and the female-biased expression of microRNAs. The female-biased expression of microRNAs can be an upstream accelerator of the cell death-related processes (i.e., cell deformation and DNA degradation) in female flower anthers. The homologues of the genes encoding these transcription factors and microRNAs are all present in the genome of female *A. officinalis* plants (12 and unpublished results of Tsugama *et al*.). The induction of *bHLH010*, *bHLH089*, *bHLH091*, *MYB33* and *MYB65* in Arabidopsis does not require *AtMYB35*^34^, and thus these genes may be induced by *M* locus genes other than *AoMYB35* (Fig. 7d). Further studies are needed to identify the upstream regulators of the expression of these transcription factor genes and microRNAs.

## Methods

### Sample preparation for RNA-Seq

Floral buds with 3–4 mm diameters, which correspond to postmeiotic stage (see Fig. 1), were collected from female plants of *the A. officinalis* cultivar New Jersey 264 and supermale plants of the cultivar Mary Washington 500 W, which had been maintained for 10–20 years in an open field of Hokkaido University. These buds were disassembled into tepals, pistils, and stamens with fine forceps and a surgical knife. The floral organs were frozen in liquid nitrogen immediately after being isolated, and then stored at −80 °C until use. Total RNA was prepared from the floral organs using a NucleoSpin RNA Plant kit (Macherey-Nagel Düren, Germany). For one replicate, ~100 buds were disassembled, and ~250 μL floral organs were used for the RNA preparation. For two biological replicates, buds were collected from different individuals on different days. Total RNA was sent to Novogene Bioinformatics Institute (Beijing, China), where mRNA purification, library preparation, and runs for RNA-Seq with NovaSeq 6000 (Illumina, Inc., San Diego, CA) were performed.

### Analysis of RNA-Seq data

Reads were obtained as 2 × 150-bp paired end reads. Reads in which more than 50% of the bases had Phred scores higher than 5 were regarded as clean reads. The clean reads were deposited in the Sequence Read Archive (SRA) in the National Center for Biotechnology Information (NCBI, https://www.ncbi.nlm.nih.gov/), and are available with the accession number SRP133335. To choose a reference for mapping, the MegaBLAST program in the NCBI BLAST+ suite^16^ was run using the RNA-Seq-derived gene sequences^8^ as queries and the *A. officinalis* genome sequence as the database. The RNA-Seq-derived gene sequences were downloaded from the Dryad digital repository^35^. The genome sequence used was generated by concatenating the 11793 nucleotide sequence records in the NCBI BioProject for *A. officinalis* genome sequencing (PRJNA317340)^12^. For the MegaBLAST analysis, default parameters were used. For mapping, two bases from the 5′ ends of the clean reads were removed using the FASTA/Q trimmer in the FASTX-Toolkit (http://hannonlab.cshl.edu/fastx_toolkit/). These trimmed reads were mapped to the RNA-Seq-derived gene sequences with Bowtie2 (version 2.3.3.1, http://bowtie-bio.sourceforge.net/bowtie2/index.shtml)^36^. For Bowtie2, the “-D 20 -R 3 -N 1 -L 20 -i S,1,0.50” option, which corresponds to the very sensitive mode allowing one-base mismatches in seed alignment, was used. With this option, approximately 90% of the reads were mapped for all the samples. The resulting Sequence Alignment/Map (SAM) files were converted to Binary Alignment/Map (BAM) files with sorted read names using SAMtools (version 1.6)^37^. Reads mapped to each gene were counted using the htseq-count program, a part of the HTSeq framework (version 0.9.1)^38^ for analyzing high-throughput sequencing data, with the “-a 0” option to count all the mapped reads regardless of alignment quality scores. The count data were managed with SQLite (version 3.9, www.sqlite.org) to obtain FPKM values for each gene and to extract SDEGs. Previously published RNA-Seq data for whole floral buds were similarly processed^10,12^. R (version 3.4.0)^39^ was used to obtain Pearson’s correlation coefficients between samples to determine hierarchical clustering of genes and samples, and to generate dendrograms and heat maps.

For GO-term and AraCyc-pathway enrichment analyses, *A. officinalis* genes with FPKM values higher than 5 and more than 5 times higher in one sex than in the other were regarded as SDEGs for each organ and each developmental stage of the flowers. For example, SDEGs for female flower pistils for the stringent analysis are the genes with FPKM values more than 5 times higher in female flower pistils than in male flower pistils. FPKM values in tepals, stamens and the other samples were not considered in this case. Jaccard indices between sets of SDEGs were obtained using R. The BLASTX program in the NCBI BLAST+ suite^16^ was run using the *A. officinalis* genes as queries and the Arabidopsis protein sequence dataset, which was downloaded from the Arabidopsis Information Resource (TAIR, https://www.arabidopsis.org/). In this analysis, approximately 38% (106284 out of 276556) of *A. officinalis* genes were related to Arabidopsis proteins with E-values lower than 1e-20. The *A. officinalis* genes and the Arabidopsis proteins were related in a one-to-one manner using the lowest E-values (<1e-20), and this one-to-one relationship was used for the analyses. Lists for GO terms and AraCyc pathways associated with each Arabidopsis proteins were downloaded from TAIR. Using these lists and the one-to-one relationship between *A. officinalis* genes and Arabidopsis proteins, the *A. officinalis* genes were associated with GO terms and AraCyc pathways. These data were managed with SQLite to obtain the numbers of genes associated with either any GO term or any AraCyc pathway (“sample sizes”) for each gene set. The numbers of genes associated with each GO term and each AraCyc pathway (“numbers of observation”) were also obtained for each gene set. The binomial approximation was applied to the relationship between the sample sizes and the numbers of observation. The ratio of the numbers of observation to the sample sizes for all the valid genes (the genes that have FPKM values higher than 5 and are associated with either any GO term or any AraCyc pathway) was used as the expected probabilities to obtain the cumulative binomial probabilities for each GO term, each AraCyc pathway and each SDEG set. Levels of significance were set as the probabilities modified by the 0.01 false-discovery rate, and were 0.00037 for the GO term analysis, and 0.000308 for the AraCyc pathway analysis. GO terms and AraCyc pathways were regarded as significantly underrepresented when their cumulative binomial probabilities were lower than those significance levels, and as significantly overrepresented when their cumulative binomial probabilities were higher than the values obtained by subtracting those significance levels from one. The exact numbers of genes associated with GO terms and AraCyc pathways and deduced cumulative binomial probabilities are given in Supplementary Tables S7 and S8. Heat maps for these analyses were generated with R.

### RT-PCR

Total RNA was prepared from floral organs of female and supermale *A. officinalis* plants as described in the “Sample preparation for RNA-Seq” subsection. First-strand cDNA was synthesized from 750 ng total RNA using the ReverTra Ace reverse transcriptase (Toyobo, Osaka, Japan) and the oligo dT_15_ primer. The resulting cDNA solutions were diluted 10 times with distilled water, and used as PCR templates. Quantitative PCR was run using these templates, the primers shown in Supplementary Table S1, GoTaq qPCR Master Mix (Promega, Fitchburg, WI), and the CFX Connect real-time PCR detection system (Bio-Rad, Hercules, CA). Relative expression levels of each gene were calculated using the comparative cycle threshold method with the *A. officinalis* gene encoding ubiquitin-ribosomal peptides fusion protein (GenBank accession number: X66875.1) as the internal control.

### Safranin-fast green staining, TUNEL staining, and Feulgen staining

For histological analyses, young floral buds were collected from female and male plants of the *A. officinalis* cultivar Ruhm von Braunschweig, which had been maintained for 10–20 years in an open field of Hokkaido University. These buds were then embedded in paraffin, semi-thin-sectioned, and deparaffinized as previously described^40^. For safranin-fast green staining, sections were incubated in 0.5% (w/v) safranin in 50% (v/v) ethanol at room temperature for 12 h, then in distilled water, 50% (v/v) ethanol, 70% (v/v) ethanol, 90% (v/v) ethanol, 99% (v/v) ethanol for several seconds each, in 0.5% (w/v) Fast Green FCF in ethanol for 3 min, in the solution of clove oil: ethanol: xylene = 2: 1: 1 (in volume) for several minutes, and in xylene for 25 min. The sections were then observed under a light microscope (ECLIPSE E600, Nikon, Co., Tokyo, Japan), and images were obtained using the CCD camera Penguin 600CL (Pixera Co., Osaka, Japan). The sections were TUNEL stained using an *In situ* Apoptosis Detection Kit (Takara Bio, Shiga, Japan). Briefly, the deparaffinized sections were incubated in a 20 μg/mL proteinase K solution for 12 min at room temperature, washed three times with phosphate-buffered saline (PBS), incubated in a terminal deoxynucleotidyl transferase-dependent DNA end labeling solution containing FITC for 30 min at 37 °C, washed three times with PBS, blocked with 1% (w/v) Block Ace (Snow Brand Milk Products Co., Ltd., Tokyo, Japan) for 10 min at room temperature, incubated in a horseradish peroxidase-labeled anti-FITC antibody solution for 30 min at 37 °C, and washed three times with PBS. Signals were then detected using a Peroxidase Stain DAB kit (Nacalai Tesque, Inc., Kyoto, Japan), the ECLIPSE E600 microscope and the Penguin 600CL CCD camera. For Feulgen staining, the sections were hydrolyzed in 5 N HCl at room temperature for 30 min, washed with distilled water, incubated in Schiff’s reagent (Merck Millipore, Billerica, MA) at room temperature for 1 h, and washed with sulfurous acid solution (Wako Pure Chemical Industries, Osaka, Japan). Signals were detected using a BX50 microscope equipped with modules for differential interference contrast microscopy and fluorescence microscopy (Olympus Co., Tokyo, Japan) and a CCD camera (ORCA-ER-1394, Hamamatsu Photonics, Hamamatsu, Japan). To quantify the signals of Feulgen staining, images were loaded into ImageJ^41^, and the “IntDen” value (“Area” × “Mean Gray Value”) for each circular signal, which corresponds to each nucleus, was obtained. Images were processed with the Canvas X software (ACD Systems, Victoria, Canada) and Inkscape (http://www.inkscape.org).

## Supplementary information

Supplementary Figures

Supplementary Table S1

Supplementary Table S2

Supplementary Table S3

Supplementary Table S4

Supplementary Table S5

Supplementary Table S6

Supplementary Table S7

Supplementary Table S8

Supplementary Table S9
