# Supplementary material for: Death of female flower microsporocytes progresses independently of meiosis-like process and can be accelerated by specific transcripts in Asparagus officinalis
Source: Sci Rep. 2019 Feb 25;9:2703. doi: 10.1038/s41598-019-39125-1 (PMC6389975; doi:10.1038/s41598-019-39125-1)
Supplement: Supplementary file 1 — Supplementary Figures [file 41598_2019_39125_MOESM1_ESM.pdf]

Death of female flower microsporocytes progresses independently of meiosis-like process and can be accelerated by specific transcripts in *Asparagus officinalis*

Mayui Ide[+], Kiyoshi Masuda[+], Daisuke Tsugama[+*] and Kaien Fujino

Laboratory of Crop Physiology, Research Faculty of Agriculture, Hokkaido University

Kita 9 Nishi 9 Kita-ku, Sapporo-shi, Hokkaido 060-8589, Japan

[+]These authors contributed equally to this work

[*]**Corresponding author:**

Daisuke Tsugama, Email: tsugama@res.agr.hokudai.ac.jp, Tel: +81-11-706-2471

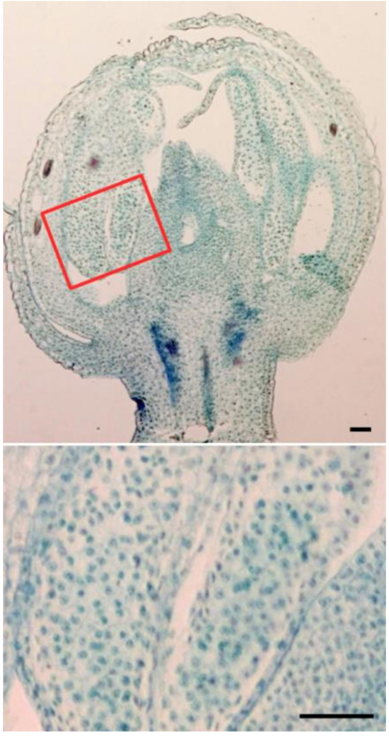

Supplementary Figure S1. TUNEL staining using an *A. officinalis* female flower at a premeiotic stage. Blue signals of the Fast Green FCF counterstaining were detected, but hardly any brown, TUNEL staining signals were detected. The bottom image is a magnified image for the region in the rectangle in the top image. Scale bars = 50 μm.

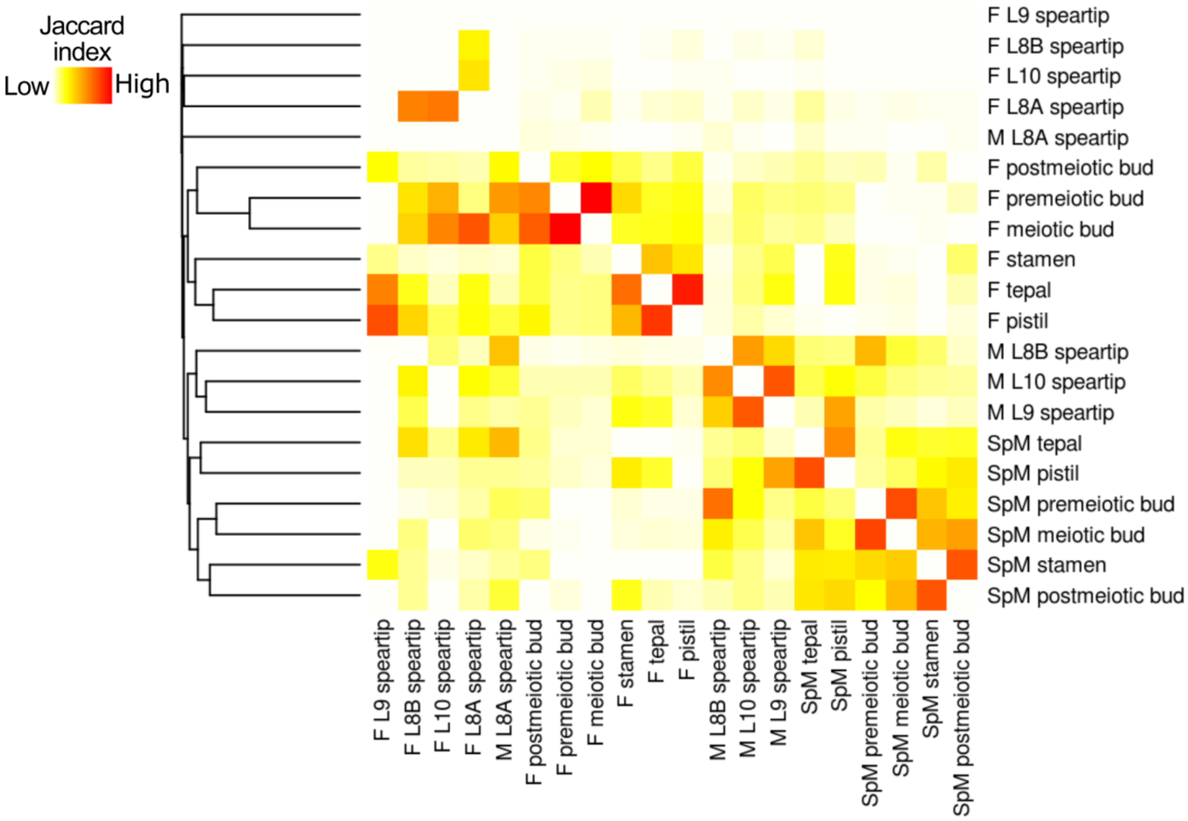

Supplementary Figure S2. Heat maps and dendrograms based on Jaccard indices for sets of SDEGs. Normalized Jaccard indices were used for coloration. Colors for the same samples (i.e., for the positions on the diagonal line) were removed for simplicity. Raw values of Jaccard indices were subtracted from 1, and the resulting values were used as distances between samples. Sample clustering was performed with the group average method[14], and the resulting dendrogram is shown in the left. F: female; M: male; SpM: supermale; L: line.

a

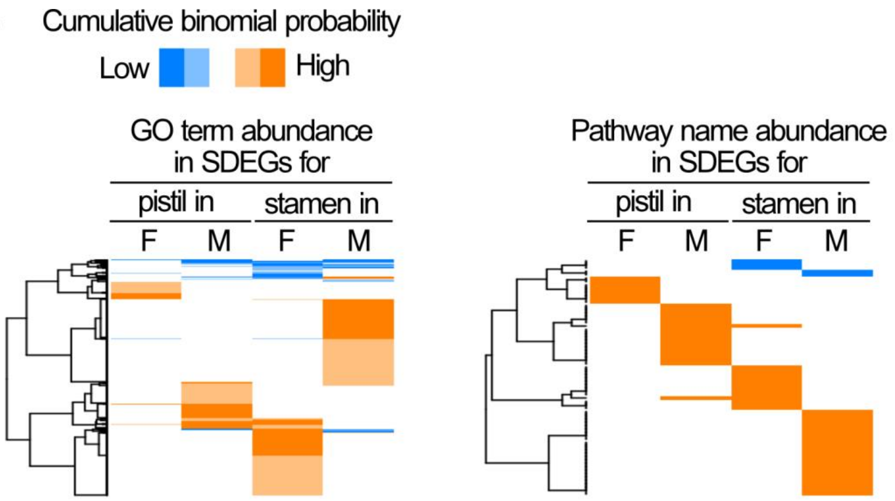

b

GO ID	Modified GO term	Abundance in all genes	Abundance in SDEGs for pistil in F	pistil in M	stamen in F	stamen in M
GO:0009555	Pollen development	1.2e-02	0	7.0e-03	4.0e-03	2.9e-02
GO:0006308	DNA catabolic process	4.2e-04	0	7.9e-03	2.9e-03	0
GO:0097468	PCD in response to ROS	1.3e-04	0	0	1.5e-03	0
GO:0009686	GA biosynthetic process	8.1e-04	0	7.9e-03	1.5e-03	2.0e-03
GO:0010372	Positive GA biosynthesis	2.6e-04	0	0	0	2.0e-03
GO:0009685	GA metabolic process	2.3e-04	0	6.2e-03	0	0
GO:0009739	Response to GA	3.7e-03	0	6.2e-03	1.1e-02	5.1e-03
GO:0009815	ACO activity	3.5e-04	0	5.3e-03	3.7e-04	0
GO:0071369	Cellular response to ET	4.8e-04	0	4.4e-03	7.3e-04	2.0e-03
GO:0010105	Negative ET signaling regulation	9.4e-04	3.5e-03	0	0	4.0e-03
GO:0009688	ABA biosynthesis	1.1e-03	0	3.5e-03	4.0e-03	0
GO:0010294	ABA glucosyltransferase activity	9.7e-05	0	1.8e-03	0	0
GO:0009733	Response to auxin	1.4e-02	3.5e-03	9.7e-03	3.3e-02	7.4e-03
GO:0000160	Phosphorelay for CK signaling	1.6e-03	0	0	6.2e-03	1.0e-03
GO:0009753	Response to JA	7.7e-03	7.1e-03	9.7e-03	2.5e-02	3.7e-03
GO:0009570	Chloroplast stroma	4.1e-02	3.5e-02	1.1e-02	4.8e-02	1.6e-02
GO:0009535	Chloroplast thylakoid membrane	1.9e-02	7.1e-03	1.8e-03	6.0e-02	4.7e-03
GO:0009522	Photosystem I	1.2e-03	3.5e-03	0	9.9e-03	0
GO:0009523	Photosystem II	1.2e-03	3.5e-03	0	8.8e-03	0
GO:0004601	Peroxidase activity	3.5e-03	1.1e-02	2.6e-03	9.2e-03	3.0e-03

c

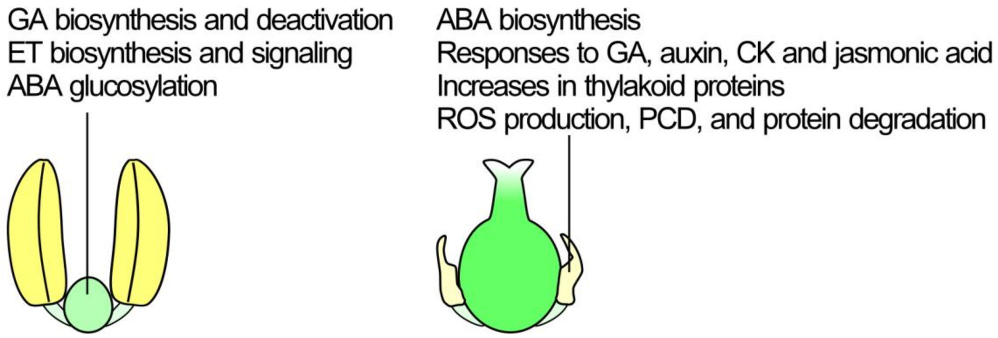

Supplementary Figure S3. GO terms and metabolic pathways associated with SDEGs. (a) Heat maps for all the GO terms and AraCyc pathway names either underrepresented or overrepresented in SDEGs for pistils and stamens. Cumulative binomial probabilities were $\log_{10}$-transformed, and used for coloration and clustering with Ward's method[15]. F: female; M: supermale (these also apply to the panel b). (b) Heat maps and abundance for selected GO terms. Some GO terms were modified for simplicity. Overrepresentation of the term "Pollen development" in SDEGs for male flower stamens is shown to validate the analysis. Values are proportions of SDEGs associated with the GO terms shown in the left. Cumulative binomial probabilities were used for coloration. (c) Schematic representation of biological processes possibly working in male flower pistils and female flower stamens.

a

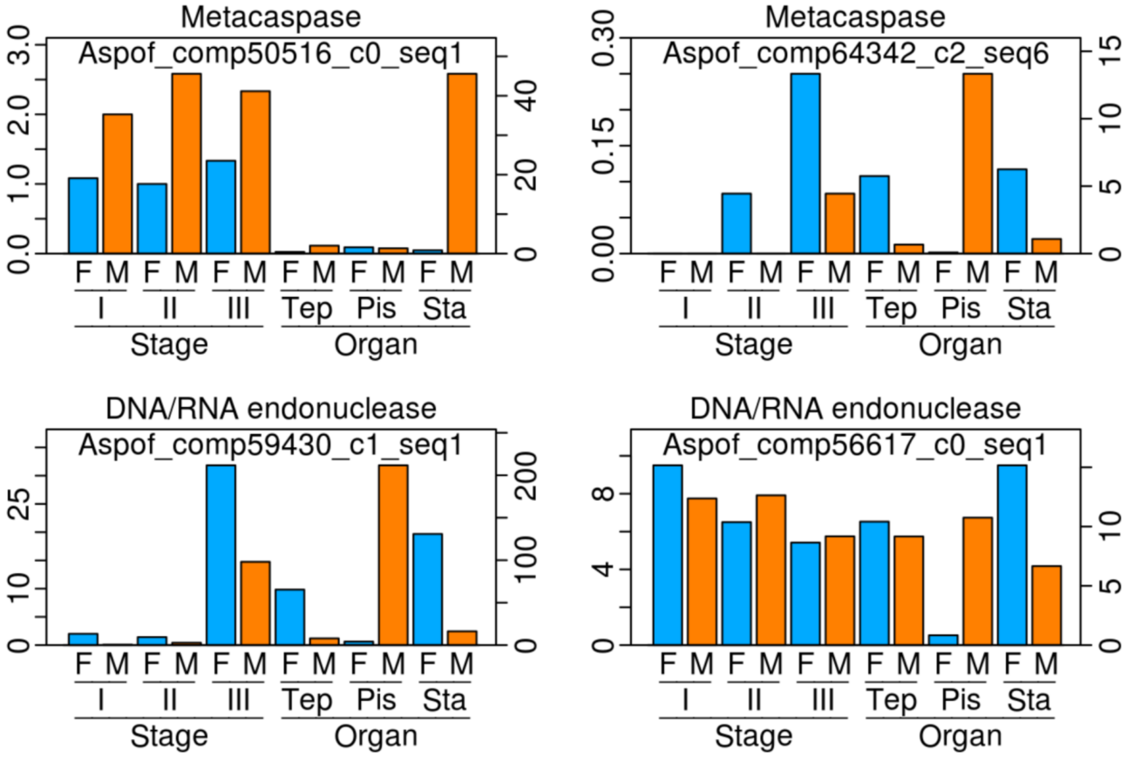
Metacaspase
Aspof_comp50516_c0_seq1
Metacaspase
Aspof_comp64342_c2_seq6
DNA/RNA endonuclease
Aspof_comp59430_c1_seq1
DNA/RNA endonuclease
Aspof_comp56617_c0_seq1
F M F M F M F M F M F M
I II III Tep Pis Sta
Stage
Organ

b

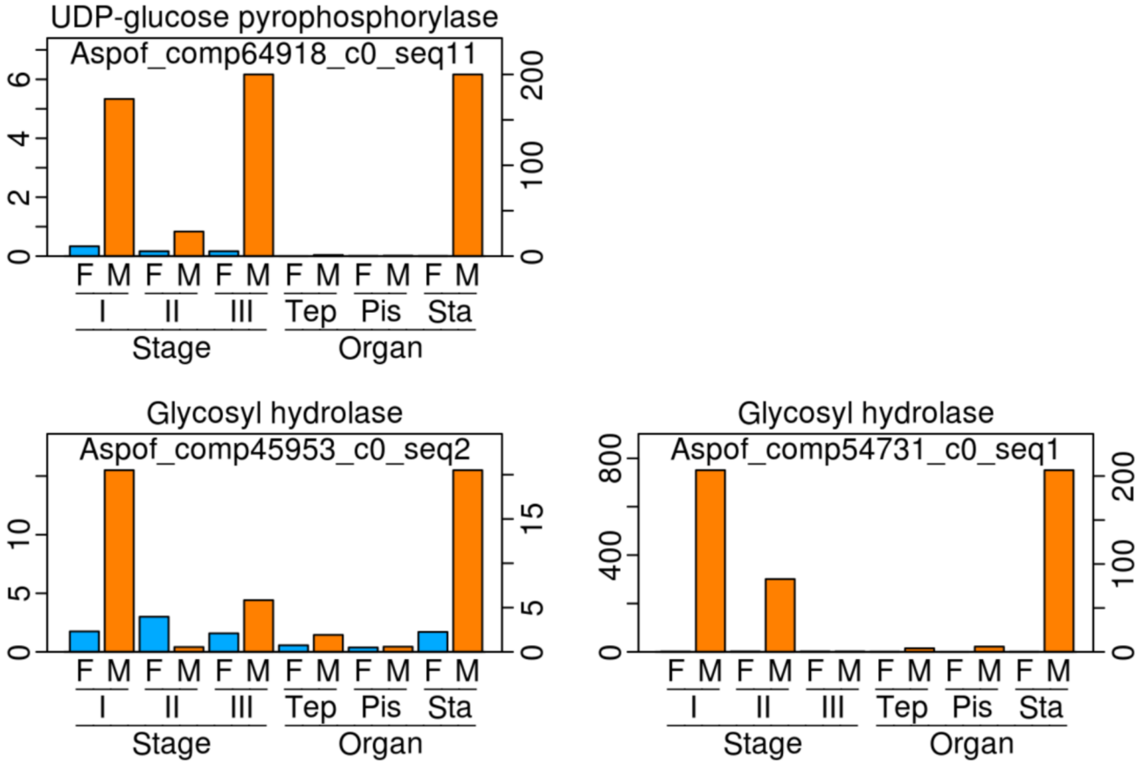
UDP-glucose pyrophosphorylase
Aspof_comp64918_c0_seq11
Glycosyl hydrolase
Aspof_comp45953_c0_seq2
Glycosyl hydrolase
Aspof_comp54731_c0_seq1
F M F M F M F M F M F M
I II III Tep Pis Sta
Stage
Organ

c

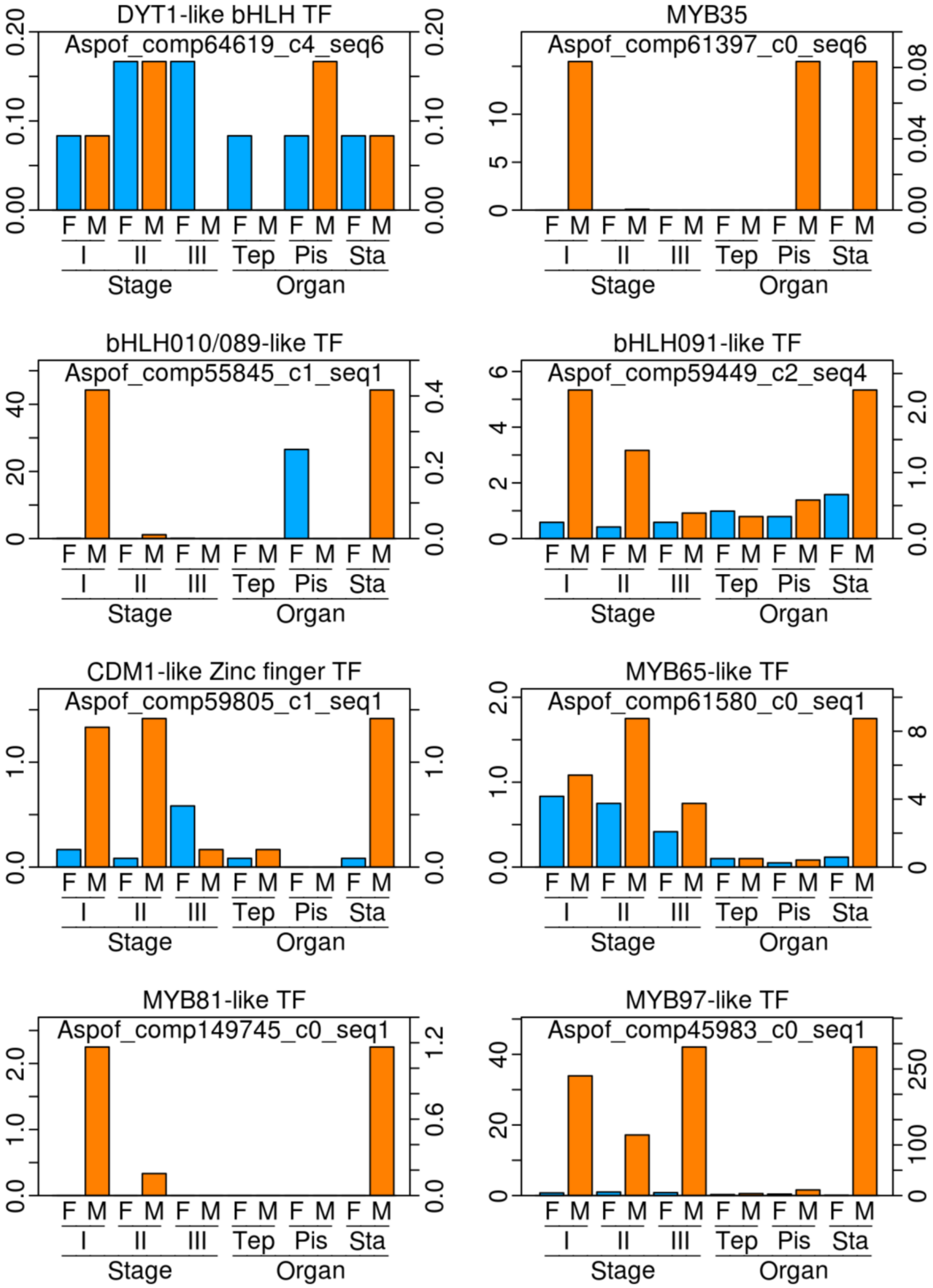
DYT1-like bHLH TF
Aspof_comp64619_c4_seq6
MYB35
Aspof_comp61397_c0_seq6
bHLH010/089-like TF
Aspof_comp55845_c1_seq1
bHLH091-like TF
Aspof_comp59449_c2_seq4
CDM1-like Zinc finger TF
Aspof_comp59805_c1_seq1
MYB65-like TF
Aspof_comp61580_c0_seq1
MYB81-like TF
Aspof_comp149745_c0_seq1
MYB97-like TF
Aspof_comp45983_c0_seq1
F M F M F M F M F M F M
I II III Tep Pis Sta
Stage
Organ

Supplementary Figure S4. FPKM values of *A. officinalis* genes possibly involved in cell death, DNA degradation (panel a), callose synthesis, its degradation (b) and transcriptional regulation (c). The FPKM values for the female (F) and male (M) flower buds at premeiotic (I), meiotic (II) and postmeiotic (III) stages were obtained using previously published RNA-Seq data[8], and are shown with the scale in the left side in each panel. The FPKM values for tepals (Tep), pistils (Pis) and stamens (Sta) in female (F) and male (M) flowers were obtained in this study, and are shown with the scale in the right side in each panel. A functional annotation indicated on the top of each panel is based on the Arabidopsis homologue most similar to the corresponding *A. officinalis* gene (see Supplementary Table S9 for accession numbers of Arabidopsis genes). TF: transcription factor.
